# The Plantaris Muscle Is Not Vestigial: Developmental, Comparative, and Functional Evidence for Its Sensorimotor Role

**DOI:** 10.3390/biology14060696

**Published:** 2025-06-13

**Authors:** Łukasz Olewnik, Ingrid C. Landfald, Bartosz Gonera, Aleksandra Szabert-Kajkowska, George Triantafyllou, Maria Piagkou

**Affiliations:** 1 Department of Clinical Anatomy, Mazovian Academy, ul. Gałczyńskiego 28, 09-400 Płock, Poland; ingridceciliee@gmail.com (I.C.L.); b.gonera@mazowiecka.edu.pl (B.G.); a.kajkowska-szabert@mazowiecka.edu.pl (A.S.-K.); 2 Department of Anatomy, Faculty of Medicine, National and Kapodistrian University of Athens, 11527 Athens, Greece; georgerose406@gmail.com (G.T.); piagkoumara@gmail.com (M.P.)

**Keywords:** plantaris muscle, anatomical variation, fetal development, tendon morphology, Achilles tendinopathy, proprioception, comparative anatomy, reconstructive surgery, muscle evolution, neuromechanical integration

## Abstract

The functional status of the plantaris muscle (PM) remains controversial and is historically dismissed as vestigial; yet, it is increasingly recognized for its structural and clinical complexity. This narrative review synthesizes current evidence from embryological development, adult morphological studies, comparative mammalian anatomy, and clinical case reports to reassess the role of the PM in humans. Developmental data reveal that the PM is consistently present during fetal life, with tendon morphology and insertion patterns emerging early and resembling adult anatomical variants. Rather than indicating postnatal regression, it suggests a stable polymorphism rooted in prenatal development. Across mammalian species, the PM varies in presence and function, correlating with locomotor specialization from proprioception in primates to propulsion in carnivores, and absence in ungulates. In humans, high proprioceptive fiber density and anatomical variability support the hypothesis that the PM may be undergoing functional repurposing from a contractile to a sensorimotor role. Clinically, its relevance is evident in imaging interpretation, surgical tendon harvesting, and the pathophysiology of Achilles tendinopathy. Recent discoveries, including the identification of the plantaris ligamentous tendon (PLT), further underscore the complexity of this region and support the need to reassess its structural and clinical significance. We conclude that the PM should not be regarded as a regressing remnant but as a dynamically adapting structure with potential neuromechanical function. Future studies involving electromyography and neuroanatomical mapping are essential to elucidate its evolving role.

## Simple Summary

The plantaris muscle (PM) has long been considered vestigial in humans, and is often described as functionally insignificant or entirely absent. However, recent anatomical, developmental, and clinical studies challenge this view. This review presents evidence that the PM is consistently present during fetal development and shows substantial anatomical variability in adults. Comparative analysis across mammals, as well as clinical case reports, suggest that the PM may have evolved from a contractile muscle to a proprioceptive and neuromechanical structure. Understanding its variations is essential for radiologists, surgeons, and anatomists, especially in the context of Achilles tendinopathy, tendon grafting, and posterior leg surgeries.

## 1. Introduction

The plantaris muscle (PM) has long occupied an ambiguous position in anatomical literature. Traditionally considered a vestigial remnant of a once-functional muscle in our evolutionary ancestors, it has frequently been regarded as functionally redundant and clinically insignificant. This assumption has been largely based on its small muscle belly, highly variable morphology, and frequent absence in the population, which is reported in up to 20% of individuals [1,2].

However, recent anatomical and developmental evidence has begun to challenge this interpretation. In a series of detailed cadaveric studies, Olewnik et al. [3,4,5,6,7] identified an unexpected degree of morphological complexity in the PM and plantaris tendon (PT), including multi-headed origins, duplicated tendons, and several previously undescribed insertion patterns. These findings suggest that the PM may not simply be degenerating, but could be functionally adaptive, or even emerging in new morphotypes rather than disappearing.

This idea is further supported by developmental observations. Waśniewska-Włodarczyk et al. [8,9] demonstrated that the PM is consistently present during fetal development, and that considerable variation in its tendon structure and insertion can already be observed in late gestation. These ontogenetic data imply that anatomical variation is likely established early in development, rather than being a postnatal regressive phenomenon.

Given these findings, the aim of this review is to critically reassess the PM by integrating data from adult human morphology, fetal development, comparative anatomy, and clinical relevance. We propose that such an integrated perspective is necessary to fully understand the form, function, and evolutionary significance of this historically overlooked structure.

### Hypothesis and Objectives

We hypothesize that the PM is not a vestigial remnant, but rather a morphologically and functionally adaptable muscle, undergoing repurposing toward a proprioceptive and neuromechanical role. This review aims to critically evaluate this hypothesis by integrating data from embryological development, adult anatomical variability, comparative mammalian anatomy, and clinical implications.

## 2. Developmental Anatomy in Human Fetuses

Understanding the ontogeny of skeletal muscles offers critical insight into their adult morphology, variability, and potential functional roles. In the case of the PM, developmental anatomy may hold the key to resolving longstanding debates surrounding its classification as a vestigial structure. Although frequently absent or morphologically inconsistent in adults, the question remains whether such variability emerges postnatally or is already encoded during prenatal development.

Recent studies examining human fetuses have provided compelling evidence that the PM forms consistently and exhibits notable variation in morphology before birth [8,9]. These observations support the notion that anatomical variation in the PM is not the result of degeneration, but rather a consequence of intrinsic developmental patterning. In this section, we explore the embryological and fetal characteristics of the PM, with a focus on its presence, origin, and PT morphology during late gestation.

To visualize the developmental dynamics of the PM during fetal life, summarizes key findings from recent embryological studies. Based on the work of Waśniewska-Włodarczyk et al. [8,9], the diagram illustrates the consistent presence of the PM belly from as early as 18 weeks of gestation, as well as the progressive increase in morphological variation in the tendon through to full-term.

The model demonstrates that PT variability reflected in differences in insertion site, trajectory, and fascial integration emerges during late gestation, supporting the hypothesis that adult anatomical variants are established prenatally. This challenges the notion that PM diversity arises postnatally, and instead suggests a developmentally encoded polymorphism.

### 2.1. Consistent Presence of the Plantaris Muscle Between 18 and 38 Weeks of Gestation

One of the central questions surrounding the PM is its classification as a vestigial structure. While its absence in adults has been reported in up to 20% of cases [1,2], prenatal investigations offer a different perspective. In a study by Waśniewska-Włodarczyk et al. [8], 80 human fetuses ranging from 18 to 38 weeks of gestation were examined, and the PM was found to be consistently present in all specimens, regardless of gestational age, sex, or crown–rump length.

The muscle belly was well-formed and distinct from the gastrocnemius muscle, displaying a typical course within the posterior compartment of the leg. No cases of agenesis or marked dysplasia were noted. These findings are particularly noteworthy in light of the hypothesis that the PM may regress postnatally or that its reported absence in adults may result from extrinsic factors such as environmental influence, epigenetic regulation, or methodological limitations (e.g., insufficient dissection technique).

In contrast to the adult anatomical literature, which emphasizes absence or high variability, the fetal presence of the PM appears to be a developmental norm rather than an exception, suggesting that the variability observed later in life arises either during postnatal development or due to diagnostic ambiguity.

These observations carry significant implications for understanding lower limb muscle development and support a re-evaluation of the PM as a potentially functional component of the musculoskeletal system, rather than a regressing remnant.

### 2.2. Tendon Insertion and Course Variability in the Fetal Period

Detailed anatomical investigations of the PM in human fetuses reveal a remarkable degree of variation in tendon morphology and trajectory that is already present during late gestation. Waśniewska-Włodarczyk et al. [9] conducted a comprehensive study on 80 fetuses aged between 18 and 38 gestational weeks (Hbd), demonstrating that although the PM was present in all cases, the structure and course of the tendon exhibited substantial interindividual differences.

Three primary patterns of insertion were identified:

1. Independent insertion into the calcaneal tuberosity, medial or slightly anterior to the Achilles tendon.
2. Fusion with the Achilles tendon, forming a shared insertion site.
3. Insertion into the deep crural fascia, without direct contact with the calcaneus.

In addition to these insertion types, variability was also observed in the tendon’s spatial trajectory. In some fetuses, the PT ran between the gastrocnemius and soleus muscles, maintaining a medial position; in others, it followed a more lateral or even oblique course before reaching its distal attachment.

Interestingly, these fetal variants strongly resemble those observed in adult cadaveric studies [6,7], suggesting that PT morphology is established during prenatal development and not the result of postnatal remodeling. These finding challenges prior assumptions that anatomical variability in the PM arises through degeneration or underuse.

Moreover, the presence of a duplicated PT was also noted in a minority of fetal specimens by Waśniewska-Włodarczyk et al. [9], mirroring adult cases described by Kurtys et al. [10]. These duplications may have functional implications, potentially influencing biomechanical tension transmission across the posterior compartment of the leg.

In summary, the PT exhibits a high degree of morphological diversity by the third trimester of gestation. This diversity appears developmentally programmed, and likely contributes to the variability seen in adult populations, as well as to the muscle’s clinical and surgical relevance.

### 2.3. The Embryological Basis of Adult Variability

Understanding the embryological development of the PM is key to interpreting its striking anatomical variability in adults. Numerous studies, including those by Waśniewska-Włodarczyk et al. [8,9], have demonstrated that the muscle and its tendon are not only consistently present during fetal development, but also exhibit a range of morphologies, including differences in origin, insertion, and trajectory, well before birth.

These findings indicate that the adult variability commonly attributed to evolutionary regression or disuse may instead be established early in ontogeny. This may result from intrinsic developmental mechanisms, such as mesodermal cell migration, local signaling pathways, or the timing of myogenic differentiation [11].

Such variations may persist into adulthood, explaining the heterogeneity seen in cadaveric and clinical studies. The consistency of these morphological variants in both fetuses and adults [5,6,8,9] supports the hypothesis that many so-called “anatomical anomalies” are in fact stable developmental variants, not degenerative features. Furthermore, the consistent emergence of accessory structures such as the plantaris ligamentous tendon (PLT), as recently described by Olewnik et al. [4], reinforces this view. The PLT develops in continuity with the superior region of the PM and exhibits a histologically distinct composition of ligamentous and tendinous fibers. Its high prevalence and well-defined architecture support the notion that such features are not random anomalies, but stable developmental offshoots with potential functional relevance [4].

This has clinical implications, particularly in diagnostics and surgical planning, where awareness of these variants can help prevent misdiagnosis or iatrogenic injury. Moreover, the apparent absence of the muscle in some adults may reflect postnatal remodeling or technical limitations in anatomical identification.

In this light, embryology offers a unifying framework for understanding PM diversity not as pathological or vestigial, but as an expression of normal developmental variation.

## 3. Adult Morphological Variability

The adult morphology of the PM is characterized by exceptional anatomical diversity, which has contributed to its ambiguous classification within both evolutionary and clinical contexts. Unlike many other skeletal muscles, the PM frequently exhibits variation in origin, insertion, and trajectory, and in some individuals, it is altogether absent [1,2,4,5,7].

Historically, this inconsistency has been interpreted as evidence of degeneration or evolutionary regression. However, mounting anatomical evidence, particularly from recent large-scale cadaveric studies, suggests that this variability may reflect stable morphological patterns rather than pathology or loss [5,7,12].

This section explores the spectrum of adult PM variation, including its prevalence, origin, insertion types, and anatomical relations, laying the groundwork for a functional and clinical re-evaluation of the muscle’s significance in modern anatomy.

### 3.1. Prevalence and Agenesis of the Plantaris Muscle

The PM is among the most anatomically variable muscles in the human body, both in terms of presence and morphology. Reports of its complete agenesis, as well as substantial differences in origin, course, and insertion, have generated debate over whether it is a vestigial structure or simply highly polymorphic.

One of the earliest large-scale anatomical studies, conducted by Daseler and Anson [1], reported the absence of the PM in 7% of 750 lower limbs. Harvey et al. [2] similarly noted its absence in 9% of their dissections. More recent data by Prakash et al. [13] indicated a higher absence rate of 26%, suggesting possible ethnic or methodological influences on prevalence rates. Other studies confirm this variability: Freeman et al. [14] and Spina [15] found rates ranging from 5% to over 20% depending on sample size, population, and anatomical technique.

In contrast, several authors have reported 100% presence of the PM in their cadaveric studies. Aragão et al. [16] and van Sterkenburg et al. [17] found the PM consistently present, highlighting that the dissection technique, population genetics, and criteria for identification may all impact findings.

Importantly, recent work by Olewnik et al. [4,5] has greatly refined the understanding of PM variability. In a series of cadaveric and imaging-based studies [5,6,7], the authors introduced detailed classifications of PM origin, PT course, insertion types, and morphological subtypes. In their research, the PM was absent in approximately 8–11% of dissected limbs. These works emphasize that what may be interpreted as agenesis could sometimes reflect fusion with adjacent structures, such as the gastrocnemius or soleus tendons, or difficulty visualizing a thin muscle belly.

Taken together, current estimates place the prevalence of PM agenesis between 7% and 20% in most populations. The discrepancy in reported prevalence likely stems from methodological diversity, inter-individual anatomical variation, and under-recognition of subtle or fused variants. Therefore, modern anatomical and clinical literature increasingly recognizes that the PM is not simply “present or absent,” but exists along a continuum of developmental and anatomical forms.

### 3.2. Classification of Plantaris Muscle Attachments

Beyond its variable presence, the PM displays remarkable morphological diversity in its proximal and distal attachments. These variations are not merely incidental findings; they hold anatomical, biomechanical, and clinical significance, particularly in surgical procedures involving the posterior knee and calf region.

Recent studies particularly those conducted by Olewnik et al. [4,5,6,7] have contributed to a structured classification of the PM based on its origin and insertion patterns. These classifications highlight the existence of distinct morphological types rather than random anatomical anomalies. The course and insertion of the PT, in particular, have been linked to pathologies such as Achilles tendinopathy and surgical complications when harvesting tendons for grafts.

Given this relevance, the following subsections explore the morphological variability of the proximal (origin) and distal (insertion) attachments of the PM, with emphasis on their frequency, anatomical relationships, and potential clinical implications.

#### 3.2.1. Proximal Attachment Variability

The PM typically originates from the lateral supracondylar line of the femur and occasionally from the oblique popliteal ligament. However, recent anatomical investigations have revealed significant variability in its proximal origin.

In a pivotal study, Olewnik et al. [5] proposed a detailed classification of proximal attachment types, based on the dissection of 142 lower limbs from adult cadavers. The authors identified six distinct origin types, reflecting the diverse anatomical possibilities of PM formation:

- Type I: divided into two subtypes:
  Subtype Ia—originates in the lateral femoral condyle, knee joint capsule, and the lateral head of the gastrocnemius muscle.
  Subtype Ib—shares the same attachments as Ia, but also includes a direct origin from the popliteal surface of the femur.
- Type II: the PM originates in the knee joint capsule and to the lateral head of the gastrocnemius muscle, which is attached indirectly to the lateral femoral condyle through the lateral head of the gastrocnemius muscle.
- Type III: originates in the lateral femoral condyle of the femur and from the knee joint capsule
- Type IV: originates in the lateral femoral condyle, knee joint capsule, and the iliotibial band.
- Type V: originates in the lateral condyle of the femur.
- Type VI: “rare cases”, all including another attachment not classified above. Within this group, two rare case reports were included:

1. Double Plantaris Muscle:

In this configuration, the main PM originates from the lateral femoral condyle, the knee joint capsule, and the iliotibial band. The muscle belly continues as a long tendon that courses between the gastrocnemius and soleus muscles. Additionally, an accessory PM originates independently from the iliotibial band. Its distal attachment fuses with the oblique popliteal ligament and connects to the direct arms of the semimembranosus tendon.

2. Bifurcated Plantaris Muscle:

This variant involves a PM with two distinct heads. The lateral head arises from the lateral gastrocnemius muscle, whereas the medial head originates from the knee joint capsule and is positioned beneath the lateral gastrocnemius. Both heads converge into a common tendon that passes deep to both the gastrocnemius and soleus muscles.

Type I was the most frequently observed pattern, present in 48.4% of specimens. The remaining types appeared with lower prevalence, emphasizing the morphological variability of the PM origin.

Olewnik et al. [5] proposed a detailed classification of eight types of PM origins based on cadaveric dissection. These types are summarized in Table 1, with descriptions and clinical relevance.

##### Comparison with Other Classifications

Nayak et al. [18] provided an early cadaveric classification of the PM origin based on 52 lower limbs from adult Indian donors. They distinguished three types of origin: the lateral supracondylar ridge, the lateral condyle of the femur, and the knee joint capsule. Additionally, they reported a rare case in which the PM originated from the fibular collateral ligament, highlighting the potential for previously underreported variants. Although not as detailed as later models, this classification contributed significantly to establishing the anatomical variability of the PM.

In contrast, Cummins and Anson [19] provided one of the earliest descriptions of PM origin variability, identifying general origin from the lateral supracondylar ridge, sometimes accompanied by fibrous extensions to the joint capsule and lateral head of the gastrocnemius. Although their system lacked formal classification, it laid the groundwork for more precise anatomical mapping.

Freeman et al. [14] offered additional observational data on PM origin, reporting that the muscle can originate from the oblique popliteal ligament and sometimes shares fibers with the lateral head gastrocnemius. Although they did not propose a classification system, their findings support the existence of accessory or blended origins, often not included in typologies such as those by Olewnik et al. [5] or Nayak et al. [18].

To contextualize the anatomical variability of the PM, a comparative overview of published origin classifications is provided in Table 2. This table highlights the evolution from descriptive observations to structured, clinically relevant typologies, culminating in the six-type classification proposed by Olewnik et al. [5].

#### 3.2.2. Distal Insertion Variability of the Plantaris Tendon

The distal insertion of the PT shows considerable anatomical variation, with clinical significance in the context of Achilles tendinopathy, tendon harvesting procedures, and posterior ankle surgeries. Modern classifications aim to document these variants in a structured, reproducible way to improve anatomical and surgical understanding.

##### Classification by Olewnik et al. [6,7]

Olewnik et al. [7] initially proposed a classification system based on a cadaveric study of 50 lower limbs, in which five primary types of the distal insertion of the PT were identified. This early typology emphasized the morphological variability of PT and its potential clinical implications, particularly in relation to midportion Achilles tendinopathy. Subsequently, the same research group conducted a more extensive anatomical study involving 130 lower limbs [6]. This investigation confirmed the previously described five types and led to the identification of a novel sixth type (Type VI). The classification, as it currently stands based on both studies, distinguishes the following six types of PT insertion:

- Type I: wide, fan-shaped insertion on the calcaneal tuberosity, medial to the Achilles tendon.
- Type II: band-shaped insertion to the Achilles tendon on the medial side, along with the Achilles tendon of the PT which was beaded in common paratenon with the Achilles tendon.
- Type III: insertion at the calcaneal bone, anterior to the Achilles tendon.
- Type IV: insertion onto the crural fascia (deep fascia of the leg), with no bony contact.
- Type V: very wide insertion encircling the posterior and medial surfaces of the Achilles tendon.
- Type VI: insertion at a point near to the tarsal canal flexor retinaculum.

The classification system for the attachment of the PT, originally proposed by Olewnik et al. [6] based on adult cadaveric material, has been further expanded by Waśniewska-Włodarczyk et al. [9], who identified two additional insertion types during a fetal anatomical study. This extended typology was subsequently confirmed in adult specimens by Patra et al. [20], reinforcing its applicability across developmental stages and its relevance to clinical anatomy.

- Type VII (added later): bifid tendon with multiple distal insertions.
- Type VIII (added later): tendon ends in the soleus muscle, with no calcaneal insertion.

This classification is considered the most comprehensive, integrating rare variants and allowing clinicians and anatomists to recognize functionally significant subtypes.

##### Comparison with Previous Classifications

Cummins & Anson [19]

This was one of the earliest documented classification systems for the insertion of the PT, based on a study of 200 lower limbs. It identified four main insertion types in relation to the Achilles tendon and calcaneal tuberosity, including insertions directly onto the calcaneus or blending with the Achilles tendon. However, this system did not account for more complex anatomical variations, such as insertions involving the crural fascia or bifid tendons. These variants were first systematically addressed in the modern classification developed by Olewnik et al. [6], which integrated both common and rare morphological patterns based on extensive cadaveric studies, offering a more clinically oriented framework.

van Sterkenburg et al. [17]

In their anatomical study focused on Achilles tendinopathy, van Sterkenburg et al. [17] proposed nine types of distal PT insertions, emphasizing clinical implications. Their types included:

1. Medial onto calcaneus
2. Medial, fan-shaped onto calcaneus
3. Medial onto calcaneal tendon
4. Medial with thin slips onto calcaneus
5. Anteromedial onto calcaneus
6. Anteromedial, fan-shaped onto calcaneus
7. Posteromedial, fan-shaped onto calcaneus
8. Anterior onto calcaneus
9. Deep fascia

This system highlighted the clinical interplay between PT variants and mid-portion Achilles tendinopathy, suggesting that certain insertion patterns may contribute to mechanical irritation or chronic pain. Unlike later classifications, such as that of Olewnik et al. [6], which provide a more structured typology based on insertion morphology and fascial involvement, the Sterkenburg system primarily focuses on topographical distribution without formal hierarchical grouping.

Dos Santos et al. [21]

Dos Santos et al. [21] proposed a simpler three-type classification based on dissection of 29 cadavers:

- Type I: the PT is inserted antero-medially into the Achilles tendon.
- Type II: the PT is inserted medially into the Achilles tendon.
- Type III: tendon merges with the Achilles tendon prior to calcaneal insertion.

Although less detailed, this classification is valuable for its emphasis on the mechanical and spatial relationships between the PT and the Achilles tendon, which are directly relevant to pathology and imaging interpretation. However, the classification proposed by Olewnik et al. [6] offers a more comprehensive and morphologically precise system, incorporating rare variants such as insertions into the crural fascia or flexor retinaculum, and thus provides greater utility for both anatomical research and clinical decision-making.

##### Clinical Significance

To enhance clarity and clinical applicability, the most relevant insertion types of the PT described across major classification systems are summarized in Table 3, highlighting their anatomical features and potential clinical implications.

Recognition of PT insertion types is essential during:

- Imaging interpretation (ultrasound, MRI), especially when assessing medial-sided Achilles pain;
- Surgical exploration, to avoid inadvertent damage to fused or accessory tendons;
- Tendon graft planning, to select appropriate tendons with predictable morphology and length.

Failure to recognize such variants may lead to misdiagnosis, mistaking PT anomalies for Achilles pathologies, or to iatrogenic complications during surgery. A thorough understanding of the full anatomical spectrum, best captured in the expanded classification by Olewnik et al. [6], is therefore crucial for anatomists, radiologists, and clinicians alike.

#### 3.2.3. Case Reports on Plantaris Muscle Variations

Although frequently dismissed as a vestigial structure, the plantaris muscle (PM) demonstrates remarkable morphological diversity. A growing body of case reports presents variations that challenge traditional anatomical models and reinforce the muscle’s potential clinical importance. These reports describe not only novel muscle origins and insertions, but also complex anatomical relations with neurovascular structures and implications for surgical practice.

##### Degenerated Accessory Head of the Plantaris Muscle

Futa et al. [22] described a rare case of the PM featuring an accessory head with evident degeneration. The main belly of the PM followed a typical anatomical course, while the accessory head originated from the iliotibial band near the distal femur. Both muscle heads merged distally into a single tendon that inserted onto the Achilles tendon. Histological examination revealed that the accessory head was markedly degenerated and composed predominantly of adipose tissue. This case highlights the clinical importance of recognizing accessory muscular components during surgical procedures involving the posterior knee and proximal calf region, as they may mimic pathological soft-tissue masses or influence surgical dissection planes.

##### Rare Neurovascular Relationship in the Popliteal Fossa

Another case by Olewnik et al. [23] described a PM with a rare spatial configuration in which the muscle’s course passed posteriorly to both the tibial nerve and popliteal vessels. This orientation poses a risk during posterior knee surgeries or diagnostic nerve blocks and supports the clinical relevance of detailed preoperative imaging in the popliteal fossa.

##### Bifurcated Plantaris Muscle with Dual Tendinous Insertions into the Iliotibial Tract and Semimembranosus

Kurtys et al. [24] reported an exceptionally rare anatomical variant of the PM, identified during routine cadaveric dissection. In this case, the PM exhibited a bifurcated muscle belly, consisting of two distinct parts: superior and inferior. Each part gave rise to a separate tendinous band, both of which demonstrated broad, fan-shaped insertions.

The superior band attached into the iliotibial tract, while the inferior band attached to the tendon of the semimembranosus muscle. No tendinous connection was observed with the calcaneus or the Achilles tendon, which is typical in standard anatomy. This configuration represents a profound deviation from the classical morphology and has not been previously documented in the literature.

From a clinical perspective, such a variant may have several implications. The presence of additional tendinous structures crossing the knee joint could be mistaken for pathological masses on imaging or during arthroscopic procedures. Furthermore, altered biomechanics at the posterolateral corner of the knee may contribute to atypical patterns of pain, particularly in cases of overuse or strain. Surgeons and radiologists should be aware of such anomalies when planning interventions in this region.

##### Three-Headed Plantaris Muscle

Olewnik et al. [25] described a rare case of a PM with three separate heads, originating independently from the posterior surface of the femur, the lateral supracondylar ridge, and the lateral head of the gastrocnemius. The bellies converged into a single tendon that inserted on the calcaneal tuberosity. This case highlights the potential for neuromuscular redundancy and challenges the notion of the PM as a uniformly vestigial muscle. The presence of multiple heads may offer enhanced proprioceptive feedback or dynamic support in the posterior compartment of the leg.

##### Three-Headed Plantaris Muscle Fused with Kaplan Fibers

A related report by Maślanka et al. [26] described a PM consisting of three distinct muscle heads, one of which was directly fused with the Kaplan fibers in the iliotibial tract. This anatomical integration, previously undescribed, could influence knee stabilization and may have relevance in orthopedic interventions involving the lateral aspect of the joint.

##### Three-Headed Plantaris Muscle with Bipartite Insertion of Its Accessory Heads

Triantafyllou et al. [27] reported a rare case of a PM variant featuring three distinct heads, all originating independently from the popliteal surface of the femur. The first head followed the typical course, contributing to a long, thin tendon that inserted onto the calcaneal tuberosity. The two accessory heads exhibited an unusual bipartite insertion: each inserted partially into the tendon of the typical head (lateral attachment) and, via musculoaponeurotic expansion, into the medial femoral condyle (medial attachment).

This is the third reported case of a three-headed PM in the literature, but the first to document this specific pattern of bipartite insertion. The variant was observed during routine cadaveric dissection and further confirmed through morphometric analysis.

From a clinical perspective, accessory muscle heads like those described may contribute to tibial nerve or vascular compression within the popliteal fossa, potentially mimicking sciatica or popliteal artery entrapment. Moreover, an awareness of such morphological complexity is essential during posterior knee surgeries or tendon harvesting procedures.

##### Four-Headed Plantaris Muscle

Zielińska et al. [12] presented the first recorded case of a PM composed of four distinct heads. Each head arose from a different anatomical structure, including the lateral femoral condyle, joint capsule, popliteal surface, and lateral gastrocnemius. All heads converged distally into a single tendon. Such a configuration could theoretically alter compartmental pressure dynamics or be misinterpreted on imaging studies as a soft-tissue mass.

##### Bilateral Variation in Origin

Hawi et al. [28] presented a case of bilateral asymmetry in the PM. On the left, two muscle bellies arose separately from the femur and merged distally, whereas on the right side, both heads shared a common origin. This variation may not present clinical symptoms, but is relevant for orthopedic surgeons and anatomists interpreting intraoperative findings or imaging.

##### Bilateral Double Plantaris Muscle with Distinct Origins and Insertions

Rana et al. [29] described an exceptional case of bilateral double PM identified during routine cadaveric dissection. In both lower limbs of a 45-year-old male cadaver, two distinct bellies of the PM were observed per side—designated as inner and outer bellies—each with separate origins, courses, and insertions.

On the right leg, the outer belly originated from the lateral lip of the linea aspera above the lateral head of the gastrocnemius and fused with the lateral side of the gastrocnemius-soleus tendon, without reaching the calcaneus. The inner belly arose from the fascia overlying the popliteus and inserted into the superior surface of the calcaneal tuberosity, crossing the tibial nerve and lying lateral to the popliteal artery.

On the left leg, the outer belly similarly arose from the lateral supracondylar ridge and merged with soft tissues between the gastrocnemius and soleus. The inner belly, thinner and more medially placed, originated from the posterior knee joint capsule and was inserted into the crural fascia just above the calcaneus. Notably, each belly received a separate branch from the nerve to the lateral gastrocnemius, indicating distinct neuromuscular control.

This is one of the very few documented cases of true bilateral double PM. From a clinical standpoint, such a configuration may have implications in posterior leg surgeries, tendon harvesting procedures, or diagnosis of soft-tissue masses in the popliteal region. Additionally, complex neural and vascular relationships necessitate caution during surgical dissection to prevent iatrogenic injury.

##### Co-Occurrence of Bilateral Plantaris Tendon Variations

In a unique cadaveric case, Olewnik et al. [30] documented two distinct PM tendon anomalies in the same individual. On the left side, the PT was inserted independently into the crural fascia without direct contact with the calcaneus, whereas on the right, the PM was completely absent. This asymmetry reflects the developmental plasticity of the PM and underlines the need for bilateral imaging when assessing posterior leg pain or planning tendon harvesting.

##### Absence of Plantaris Tendon with Fascial Continuation into the Soleus Muscle

Sugavasi et al. [31] described a unique anatomical variation in which the PM was present but lacked a distinct tendon. The PM originated from the lateral supracondylar line of the femur and the oblique popliteal ligament, as is typical. However, instead of forming a free tendon, the distal portion of the muscle continued as a flat fascial sheet, which merged directly into the fascia of the soleus muscle. This variant was observed unilaterally in the right lower limb of the cadaver.

From a clinical perspective, the absence of a discrete PT can pose challenges during autologous tendon harvesting, commonly performed in Achilles tendon repair or reconstructive surgeries. This case highlights the importance of preoperative imaging and careful intraoperative assessment to avoid confusion or surgical failure when the tendon is assumed to be present but is in fact absent or modified.

##### Morphological Variation Affecting Tendon Harvesting

In a surgically relevant case, Gonera et al. [32] reported a PM tendon that inserted onto both the medial aspect of the Achilles tendon and the crural fascia. This dual insertion could complicate tendon harvesting procedures, particularly when the plantaris is used as an autologous graft. Awareness of such dual insertions can help prevent harvesting failures or complications.

##### Highly Complex Multiband Plantaris Tendon Insertion

Kurtys et al. [10] described a previously unreported and highly intricate variant of the PT insertion observed during dissection of a 68-year-old male cadaver. The PT demonstrated four separate bands of distal attachment to the calcaneus and a tendinous bridge to the Achilles tendon, making it one of the most morphologically complex plantaris variants described in the literature.

The tendon was divided into the following insertional structures:

- Band A1: a broad, fan-shaped insertion on the superomedial calcaneal tuberosity.
- Band A2: a medial band inserting lower on the calcaneal shaft.
- Band B1: an anterior band positioned near the anterior Achilles insertion.
- Band B2: an inferomedial band inserting onto the mid-shaft of the calcaneus.
- Band C: a tendinous connection extending from Band A to the medial margin of the Achilles tendon.

This highly complex multiband insertion pattern may increase the risk of mechanical conflict or traction-related irritation in the posterior ankle, especially during dynamic loading. Additionally, such intricate morphology could complicate tendon harvesting procedures or be misinterpreted as pathological structures in imaging.

##### Clinical Implications

Collectively, these case reports underscore several key themes:

- Developmental complexity: the PM may present with multiple heads, duplications, or complete agenesis, often bilaterally asymmetrical.
- Functional integration: fusion with nearby structures (e.g., Kaplan fibers, Achilles tendon) indicates that the PM may still serve a biomechanical or proprioceptive role.
- Surgical relevance: variant insertions affect tendon graft usability, dissection complexity, and potential neurovascular entrapment.

These findings support a reconceptualization of the PM as a variable, potentially functional muscle rather than a vestigial remnant.

### 3.3. Topographic Relationships

The PM and its long tendon are located within the posterior compartment of the leg, and their spatial relationships to adjacent neurovascular and musculoskeletal structures are of critical anatomical and clinical interest. Topographic variability may influence surgical approaches to the knee and ankle, as well as the development of compressive neuropathies or tendinopathies.

#### 3.3.1. Relationship to the Achilles Tendon

The most clinically relevant anatomical relationship of the PT is with the Achilles (calcaneal) tendon. Two primary variants of its course in relation to the Achilles tendon were described by Olewnik et al. [6,7]:

- Variant A: the PT runs between the gastrocnemius and soleus muscles, then travels medially to the Achilles tendon before inserting onto the calcaneus.
- Variant B: the PT emerges similarly but proceeds anterior to the Achilles tendon, sometimes crossing over it medially before insertion.

This close medial or anteromedial relationship between the PT and the Achilles tendon is believed to be a contributing factor in the development of mid-portion Achilles tendinopathy, particularly in patients with pain localized to the medial aspect of the Achilles tendon. During plantarflexion under load, the PT may exert compressive or shearing forces on the Achilles tendon, potentially leading to irritation, microtrauma, or chronic inflammation [17,33,34].

Variability in the course of the PT relative to the Achilles tendon plays a potentially significant role in the development of Achilles tendinopathy. In particular, Variant A, where the PT runs parallel and medial to the Achilles, may create a zone of mechanical interference that exacerbates friction, compression, or tension along the medial border of the Achilles. Previous studies have shown that in approximately 41% of cases, the PT lies close to the medial aspect of the mid-portion of the Achilles tendon, a location commonly associated with pain in chronic tendinopathy [7,17,34].

Variant B, though less common, may also be clinically relevant. Its anterior course relative to the Achilles may increase localized compression forces, especially during dynamic ankle movement or tendon loading. This could contribute to inflammation, microtears, or chronic pain, especially in active individuals.

Understanding these anatomical variations is critical for accurate diagnostic imaging, effective conservative management, and successful surgical intervention. High-resolution ultrasound and MRI should account for these anatomical variants to prevent misdiagnosis or overlooking a mechanically significant plantaris tendon.

From a surgical perspective, knowledge of the PT course may influence decisions regarding tendon excision, Achilles tendon debridement, or reconstructive graft harvesting. Tendon surgeons must remain aware of potential anatomical variants to minimize complications and optimize patient outcomes.

#### 3.3.2. Relationship to Other Musculotendinous Structures

The PT typically passes deep to the gastrocnemius and superficial to the soleus; however, variations may occur. In some cases, the tendon is entirely embedded within the muscle belly of the soleus or runs unusually superficial to the gastrocnemius. These positional anomalies can affect:

- The visibility of the PT on ultrasound, particularly in thin or muscular patients;
- The ease of PT identification and isolation during harvest procedures;
- The surgical dissection plane in open or arthroscopic interventions in the calf and ankle.

In some reported cases, the PT merges with, or even partially replaces, fascicles of the Achilles tendon [18,21].

#### 3.3.3. Clinical Significance

Awareness of PM topography is essential during:

- Posterior knee approaches, especially for ligament reconstruction;
- Tendon graft harvesting, to prevent incomplete or inadequate graft collection;
- Differential diagnosis of posterior leg pain, particularly in cases of chronic medial-sided pain.

Modern imaging techniques such as high-resolution ultrasonography and MRI have proven effective in identifying these variations preoperatively. Nonetheless, surgeons must remain aware of the high degree of individual anatomical variability and adjust their techniques accordingly.

## 4. Comparative Anatomy Across Mammals

The PM, although variably present in humans, exhibits considerable interspecies morphological diversity among mammals. In many quadrupeds, the PM plays a key role in propulsion and plantarflexion, whereas in humans and other primates, its function is less well defined and often considered vestigial [35].

Comparative anatomical research suggests that the muscle’s form and function are tightly linked to locomotor behavior and postural demands, reflecting both evolutionary retention and reduction depending on species-specific adaptations [36,37,38]. As such, the PM serves as a useful model for studying muscle regression, redundancy, and neuromuscular adaptation.

In the following sections, we review the presence, morphological variants, and potential functions of the PM across a representative selection of mammalian species and explore its phylogenetic implications.

### 4.1. Presence of the Plantaris Muscle Across Mammalian Orders

The presence, structure, and functional relevance of the PM vary widely across mammalian taxa. These differences are often shaped by locomotor behavior, ecological niche, and evolutionary lineage. While the PM is sometimes absent or vestigial in humans, it remains robust and biomechanically important in many non-primate mammals.

#### 4.1.1. Primates

In humans (*Homo sapiens*), the PM is inconsistently present, with agenesis reported in approximately 7–20% of individuals [1,6]. Even when present, the muscle is often small and contributes minimally to force generation during plantarflexion. However, several authors have suggested a possible proprioceptive role due to its high density of muscle spindles relative to its size [14,15] (Freeman et al., 2008; Spina, 2007).

In non-human primates, such as chimpanzees (*Pan troglodytes*), the PM is more consistently present and often more robust in terms of size and anatomical distinction from the gastrocnemius. Its presence is thought to correlate with greater arboreal activity and the demand for strong ankle flexion during vertical climbing [39]. In arboreal species like macaques (*Macaca* spp.), the PM remains well-developed and functionally distinct, whereas in more terrestrial primates, including some baboons, partial fusion with the gastrocnemius or reduction in size is more commonly observed [40].

These anatomical patterns support the hypothesis that PM development reflects functional demand and locomotor behavior, particularly in the context of climbing and grasping, where fine and independent control of the ankle joint is advantageous.

#### 4.1.2. Carnivores

Among carnivores, the PM is typically well-developed and functionally integrated within the common calcaneal tendon complex. In dogs (*Canis familiaris*) and cats (*Felis catus*), it contributes significantly to extension of the tarsal joint and propulsion during running or leaping [41].

Anatomically, the PM originates from the lateral supracondylar region of the femur and courses distally to join the common calcaneal tendon, alongside contributions from the gastrocnemius, superficial digital flexor, and other muscles. Its long, tendinous structure acts as an elastic energy store, assisting in efficient limb recoil and stride economy—key adaptations for cursorial and saltatory locomotion [41,42].

#### 4.1.3. Ungulates (Hoofed Mammals)

In ungulates such as horses (*Equus caballus*), cattle (*Bos taurus*), and sheep (*Ovis aries*), the PM is either highly reduced or completely absent [43]. In horses, the PM is typically fused with or replaced by the superficial digital flexor and gastrocnemius muscle–tendon complex, reflecting an evolutionary emphasis on long tendinous structures that optimize elastic recoil and enhance efficiency during cursorial locomotion.

The near or total absence of the PM in these species exemplifies evolutionary streamlining, whereby distal limb musculature is minimized to reduce limb mass and energetic cost in fast-moving quadrupeds. These animals rely heavily on passive elastic elements such as tendons and ligaments for locomotor efficiency, with minimal reliance on small accessory muscles like the PM [42,43].

#### 4.1.4. Functional and Evolutionary Summary

The degree of development and independence of the PM across mammals provides important insights into its functional plasticity:

- In arboreal and climbing species (primates), the PM may assist with fine-tuned ankle control [39].
- In cursorial species (carnivores), it contributes to propulsion and energy storage [41].
- In highly specialized runners (ungulates), it is largely regressed or integrated into other muscle–tendon systems [42,43].

This variability supports the interpretation of the PM as a modular muscle, one that is evolutionarily retained, reduced, or lost depending on biomechanical needs rather than being universally vestigial. Its morphology in any given species reflects an intricate interplay between evolutionary heritage, functional redundancy, and ecological pressures [1,5].

To better illustrate the phylogenetic and functional variability of the plantaris muscle across mammalian orders, Table 4 summarizes its presence, absence, or variability in selected representative species. This comparative overview reflects the relationship between plantaris development and locomotor specialization, highlighting the evolutionary significance of its retention in some taxa and loss in others.

#### 4.1.5. Comparative Anatomy Based on Barone

The detailed comparative anatomy of the plantaris muscle across domestic mammals has been comprehensively described by Barone [44] in his seminal work *Comparative Anatomy of Domestic Mammals*. Barone’s observations provide important insights into the morphological variability and functional adaptations of the plantaris muscle within various species.

In the domestic dog (*Canis familiaris*), the plantaris muscle is well-developed, originating from the lateral supracondylar ridge of the femur and inserting into the calcaneal tendon, where it contributes significantly to tarsal extension and propulsion during locomotion. Its long, slender tendon serves as an elastic element facilitating efficient energy storage and release during the gait cycle. [44]

Conversely, in ungulates such as the horse (*Equus caballus*), cattle (*Bos taurus*), and sheep (*Ovis aries*), Barone noted that the plantaris muscle is often reduced, rudimentary, or entirely absent. When present, it may be fused with neighboring muscles like the superficial digital flexor or gastrocnemius, reflecting evolutionary modifications aimed at reducing distal limb muscle mass for enhanced locomotor efficiency. This trend exemplifies the functional and ecological specialization observed in cursorial mammals [44].

Furthermore, Barone highlighted interspecies differences in the origin, insertion, and tendon morphology of the plantaris muscle, emphasizing its developmental plasticity and adaptive capacity. For instance, the plantaris in domestic cats (*Felis catus*) is relatively more prominent and distinct compared to larger ungulates, correlating with their unique locomotive behaviors and biomechanical demands.

Incorporating Barone’s [44] detailed descriptions enriches the understanding of plantaris muscle evolution and supports the hypothesis that its anatomical variability reflects functional specialization rather than simple vestigial regression.

### 4.2. Function and Regression of the Plantaris Muscle

The functional relevance of the PM remains a subject of debate. While some regard it as a vestigial structure in humans, its persistence across numerous mammalian taxa and frequent anatomical complexity suggest otherwise. Across species, the PM may fulfill roles in proprioception, plantarflexion, and tendon load modulation functions that are retained or lost depending on evolutionary and biomechanical context [5].

#### 4.2.1. Grasping and Supportive Function in Primates

In arboreal primates, such as macaques, colobus monkeys, and chimpanzees [39], the PM is typically more robust than in humans. It is structurally distinct from the gastrocnemius and likely supports grasping, ankle stabilization, and climbing. These species often rely on flexed limb postures, where ankle control is critical.

#### 4.2.2. Proprioceptive Capacity

Histological studies indicate a high density of muscle spindles in the human PM [14,15], suggesting a proprioceptive role. This could involve feedback during eccentric Achilles loading or subtle postural adjustments. In species like dogs and cats, where the PM is part of the calcaneal tendon complex, it may serve both proprioceptive and mechanical functions [41].

#### 4.2.3. Evidence of Regression in Humans

Despite variability and frequent agenesis [1,6], the human PM may persist for sensory rather than motor roles. Its limited mechanical contribution supports theories of functional repurposing, not redundancy [5,15]. This would explain evolutionary retention despite anatomical downsizing. Furthermore, the presence of novel, possibly functionally adaptive structures such as the PLT in humans—which are absent in most quadrupedal species—may represent a specific adaptation to bipedal locomotion. The PLT originates from the superior portion of the PM and connects to the iliotibial band, suggesting a unique mechanical role in lateral knee stabilization. Its discovery not only underscores the morphological complexity of the plantaris complex, but also supports the idea that human evolution may favor structural repurposing over regression [4].

#### 4.2.4. Vestigiality and Functional Redundancy

In bipedal humans, the demand for fine ankle manipulation and powerful plantarflexion is largely met by the gastrocnemius, soleus, and tibialis posterior. The PM, when present, exhibits a narrow fusiform belly and a long, slender tendon that contributes little to force production [2,45]. In some cases, the muscle is entirely fused with the gastrocnemius or even misidentified during surgery [5]. These features, coupled with high rates of anatomical variability, support the theory that the PM is in evolutionary decline, especially when compared to its more robust form in arboreal or cursorial mammals [39].

#### 4.2.5. Proprioceptive Role in Humans

However, emerging evidence suggests that the PM may retain neurological relevance despite its mechanical redundancy. Histological studies have shown a disproportionately high density of muscle spindles in the PM, suggesting a role in proprioceptive feedback [14,15]. This sensory function could explain its evolutionary persistence: rather than contributing directly to locomotion, the PM may monitor tension or position changes within the Achilles tendon complex. Such a mechanism would be especially important during eccentric loading in running or stair descent, where dynamic stabilization and feedback are essential.

Additionally, the anatomical relationship between the PT and Achilles tendon in some individuals particularly when they are closely apposed or fused may support a sensory integration role, whereby the PM contributes to a broader mechanosensory network for ankle joint function [5,6].

### 4.3. Phylogenetic Tree of Mammals

**Figure 1:**
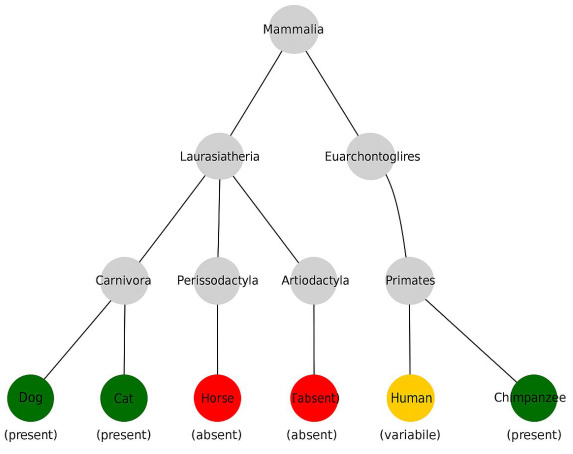
Phylogenetic tree of mammals with plantaris muscle presence indicated.

The diagram (Figure 1) illustrates the phylogenetic relationships between major mammalian groups, with node color indicating the status of the plantaris muscle in representative species:

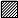
**Green**—plantaris muscle consistently present;
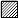
**Orange**—plantaris muscle variably present or vestigial;
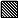
**Red**—plantaris muscle absent or regressed.

Taxa include primates (*Homo sapiens*, *Pan troglodytes*), Carnivora (*Canis familiaris*, *Felis catus*), Perissodactyla (*Equus caballus*), and Artiodactyla (*Bos taurus*). Phylogenetic topology is based on Meredith et al. [46] and Murphy et al. [47].

## 5. Functional Role of the Plantaris Muscle

Although historically considered a vestigial remnant in humans, the PM exhibits a spectrum of functional roles across vertebrate species and even within human populations. Recent anatomical, histological, and biomechanical studies suggest that the PM may contribute to proprioception, joint stabilization, and tendinous elasticity, rather than acting as a primary mover in locomotion [5,14,15,17,34].

This section explores the potential functional contributions of the PM in both evolutionary and clinical contexts. Through analysis of muscle fiber composition, neurosensory innervation, and its biomechanical relationship to the Achilles tendon, we aim to determine whether the PM should be viewed as a declining anatomical vestige or a specialized component of the posterior leg compartment.

### 5.1. Histological and Biomechanical Evidence

Although often overlooked due to its small size and anatomical variability, the PM may possess specialized histological features that hint at a persistent neuromuscular function. Key among these is its high density of muscle spindles, which suggests a role in proprioception rather than force generation.

Several histological analyses have demonstrated that the PM, particularly in humans, contains a disproportionately high number of muscle spindles per gram of tissue compared to adjacent calf muscles such as the gastrocnemius and soleus [15,48]. This dense concentration of intrafusal fibers implies that the PM functions as a sensory organ, providing feedback on muscle length and tension during movement. Freeman et al. [14] noted that the PM might serve as a proprioceptive monitor of the Achilles tendon, particularly in conditions of eccentric loading or sudden changes in ankle position. This function is especially relevant during activities such as stair descent, sprinting, or balance correction.

Biomechanically, the PT is long, thin, and elastic, often running in close proximity to or even fusing with the Achilles tendon. This configuration suggests that it may act as a modulator of tendon tension or a secondary elastic component in the posterior compartment of the leg [17,34]. Although the muscle belly is weak, its long tendon may still transmit small corrective loads or play a stabilizing role during dynamic movements. Furthermore, in individuals with variant insertions such as direct fusion with the Achilles tendon or broad fascial attachments the PM may alter the load distribution in the Achilles complex. This could have implications for injury susceptibility, especially in tendinopathy-prone athletes [6].

### 5.2. Influence on the Knee and Ankle Joint

Although not a major contributor to joint movement, the PM may affect both mechanical dynamics and proprioception in the knee and ankle.

The PM often originates from the lateral supracondylar ridge and joint capsule [5]. Fascial connections with the posterior capsule suggest a role in stabilizing the knee and sensing joint angle or tension [12,49]. Its posterolateral position is well-suited for contributing during extension and eccentric movements.

The PM tendon typically inserts near or into the calcaneus, often near the Achilles tendon. It may modulate force during plantarflexion, transmit proprioceptive signals, and stabilize the subtalar joint. In cases of Achilles tendinopathy, PM variants may be clinically relevant due to friction or mechanical overlap [6,17,34]. These subtle roles support the view of the PM as a neuromechanical integrator in the lower limb.

### 5.3. Intramuscular Innervation and Neuromuscular Control

Recent neuroanatomical investigations using a modified Sihler’s staining technique have revealed that the PM exhibits a rich and organized intramuscular innervation pattern [3]. Multiple distinct nerve entry points were observed, predominantly from the medial aspect, with fine arborizing branches extending throughout the muscle belly. In addition, the location of motor endplate zones suggests a distributed activation system that supports fine neuromuscular control rather than gross motor function [3].

These findings align with the high density of muscle spindles previously identified histologically, reinforcing the concept that the PM may function primarily as a sensorimotor integrator. The detailed intramuscular branching pattern indicates active participation in proprioceptive feedback mechanisms, particularly in relation to tension monitoring within the posterior leg compartment and potentially the Achilles tendon [3].

As such, the innervation architecture of the PM further strengthens the argument that this muscle should not be dismissed as vestigial, but rather acknowledged as functionally relevant in neuromechanical regulation.

### 5.4. Arguments Against the “Vestigial Muscle” Theory and Emerging Perspectives on Functional Development

The PM has long been described as vestigial in humans a nonfunctional remnant of our quadrupedal ancestry. This perspective has been shaped by its small size, high variability, and limited contribution to ankle plantarflexion. However, an increasing number of anatomical and developmental studies challenge this view, suggesting that the PM may be functionally repurposed rather than regressing.

#### 5.4.1. Consistent Presence in Fetal Development

Waśniewska-Włodarczyk et al. [8,9] showed that the PM is consistently present during late fetal development (18–38 weeks), often with well-formed muscle bellies and distinctive tendons. This regularity argues against a random or degenerative process and instead suggests that agenesis in adults may reflect secondary postnatal factors, such as atrophy or misclassification.

#### 5.4.2. Complex Morphological Variants and Active Remodeling

Numerous reports have documented complex variants of the PM including three- and four-headed muscles, bifid tendons, and intertendinous fusion with the Achilles tendon [10,12,23,24,25,26,27,30,32,50]. These findings are inconsistent with anatomical regression and instead suggest ongoing morphological plasticity. Critically, Olewnik et al. [5] have explicitly questioned the vestigial status of the PM in modern humans, proposing that it may, in fact, represent a muscle undergoing functional and anatomical development. In their view, the PM may be in the process of acquiring a new functional role, particularly in proprioception and sensorimotor integration.

#### 5.4.3. Proprioceptive Capacity

As highlighted in Section 5.1, the PM demonstrates an unusually high density of muscle spindles well above that of surrounding muscles implying a strong sensory function [15,48]. This histological profile is characteristic not of regressing muscles but of those that retain an important neurosensory role in joint monitoring and coordination.

#### 5.4.4. An Emerging Hypothesis: The Plantaris as an Adaptive Muscle

Rather than disappearing, the PM may be evolving toward a specialized proprioceptive and stabilizing role. This view aligns with broader trends in muscle evolution, in which structural downsizing accompanies a functional shift from motor to sensory contributions. This would classify the PM as a modular muscle, adapting to reduced mechanical load by enhancing neurosensory feedback [39]. Supporters of this view argue that PM morphology reflects not loss, but transition a shift in anatomical relevance from power generation to fine control of movement and force sensing, particularly in complex bipedal locomotion.

#### 5.4.5. The Plantaris Ligamentous Tendon

A novel anatomical structure known as the *plantaris ligamentous tendon* (PLT) has recently been described by Olewnik et al. [4], further challenging the long-standing classification of the PM as a vestigial structure. The PLT was identified in 72.7% of the lower limbs examined and originates from the superior part of the PM, the posterior surface of the femur, and the lateral aspect of the knee joint capsule, inserting into the iliotibial band. Histological analysis demonstrated the coexistence of tendon-like and ligament-like fiber architectures within the same structure, indicating a hybridized, biomechanically adaptive entity [4].

Clinically, the location and morphology of the PLT suggest a possible role in the development or exacerbation of iliotibial band syndrome, a common overuse injury characterized by lateral knee pain, especially in runners and cyclists. The attachment point of the PLT—approximately 1–3 cm superior to the lateral joint line—corresponds precisely with the typical area of tenderness in ITBS. Its potential to increase fascial tension or friction over the lateral femoral epicondyle may contribute to symptom generation [4].

From a surgical and diagnostic perspective, unawareness of the PLT’s existence could lead to misinterpretation during imaging or inadvertent damage during procedures involving the posterolateral knee. Its histological distinction from accessory bands of the PT is critical for anatomical classification and future surgical relevance.

The identification of the PLT reinforces the growing recognition that the PM complex is neither functionally obsolete nor structurally regressing. Instead, it may represent a dynamic and evolving sensorimotor unit. Future studies should explore its involvement in lateral knee pathologies and biomechanical function through imaging, cadaveric simulation, and electromyographic correlation.

Conclusion: Collectively, these findings suggest that the PM should not be universally regarded as a vestigial structure. Instead, in light of developmental consistency, structural complexity, and proprioceptive capacity, it may be more accurately described as a functionally transforming muscle one that continues to evolve new roles in human locomotor adaptation.

## 6. Clinical Significance

Once considered functionally redundant, the PM has gained renewed clinical interest due to its involvement in several pathological and surgical contexts. Anatomical studies have demonstrated significant variability in its morphology, trajectory, and tendon insertion, which may influence surgical outcomes and contribute to diagnostic errors, particularly in the posterior knee and ankle regions [6,7,18].

Clinically, the PT has been implicated in midportion Achilles tendinopathy, particularly when it courses in close proximity to or fuses with the Achilles tendon, potentially altering local biomechanics and generating irritation [51,52]. Furthermore, the PT is frequently utilized as an autograft in tendon reconstruction procedures, such as Achilles repair, flexor tendon reconstruction, and facial reanimation, owing to its anatomical properties and low donor-site morbidity [53,54,55].

This section discusses the complex clinical relevance of the PM, highlighting its role in injury, imaging, reconstructive surgery, and rehabilitation strategies.

### 6.1. Injuries and Pathologies of the Plantaris Muscle

Although often underestimated, the PM may contribute to calf pain syndromes, particularly among athletes.

#### 6.1.1. Tennis Leg and Rupture

Traditionally associated with medial gastrocnemius strain, tennis leg may also involve isolated or combined rupture of the PM [56]. Magnetic resonance imaging (MRI) often reveals hematoma and tendon retraction between the gastrocnemius and soleus muscles [57], supporting the need for differential diagnosis.

#### 6.1.2. Diagnostic Challenges

Due to its variability, the PM may be misinterpreted as an accessory muscle, venous pathology, or may be completely overlooked during surgical procedures [58,59]. Anatomical variants such as tendon duplication, fusion with the Achilles tendon, or fascial insertions must be recognized to avoid misdiagnosis or iatrogenic injury [4,6].

#### 6.1.3. Clinical Significance

Accurate identification of the PM is essential for proper diagnosis, to avoid unnecessary treatments (e.g., anticoagulation in misdiagnosed deep vein thrombosis), and for planning appropriate rehabilitation strategies. While not life-threatening, unrecognized PM ruptures may lead to delayed return to activity and chronic discomfort [60].

### 6.2. Reconstructive Surgery and the Use of the Plantaris Tendon

The PT is an increasingly popular graft option in various reconstructive surgeries due to its length, thin diameter, and minimal impact on limb function following harvest [21,61]. The tendon is accessible through a small posterior leg incision and demonstrates biomechanical properties comparable to native tendons.

#### 6.2.1. Tendon Grafting Applications

1. Achilles Tendon Repair

The PT is commonly used in the reconstruction of chronic or degenerative Achilles tendon ruptures. It serves to bridge large defects or reinforce weakened areas following re-rupture. Techniques such as the turndown graft or loop weaving improve tensile support and minimize gapping during healing [51,53].

2. Hand and Finger Tendon Reconstruction

With an average length exceeding 30 cm, the PT is particularly suited for flexor tendon grafting in cases of irreparable digital injuries. It offers favorable gliding and integration characteristics, as reported by Bunnell [62] and confirmed in microsurgical practice [61].

3. Facial Reanimation and Esthetic Surgery

In dynamic and static reanimation for facial paralysis, the PT has been employed as a tensioning graft due to its elasticity and favorable esthetic outcomes. Its use minimizes visible scarring and functional asymmetry [63].

#### 6.2.2. Advantages and Considerations

The advantages of the PM include the following:

- Minimally invasive harvest;
- Minimal donor-site morbidity;
- Adequate length and mechanical resilience;
- Biomechanical similarity to flexor tendons.

Nevertheless, anatomical variation, including agenesis, duplication, or fusion with Achilles tendon, necessitates careful preoperative planning. Imaging modalities such as ultrasound, MRI, or intraoperative inspection, are advised to confirm graft suitability [6,21].

### 6.3. Imaging Relevance in Differential Diagnosis of Achilles Tendon Injuries

The close anatomical relationship between the PT and the Achilles tendon presents a diagnostic challenge, particularly in patients presenting with posterior leg pain or suspected Achilles tendinopathy. Imaging, especially high-resolution US and MRI, plays a pivotal role in identifying PT variants and differentiating them from true pathological findings [51,52,64].

#### 6.3.1. Ultrasound (USG) Evaluation

Ultrasonography is a cost-effective, dynamic, and real-time modality that enables detailed visualization of soft-tissue structures in the posterior leg. It is particularly useful in assessing the following:

- The spatial relationship of the PT relative to the medial Achilles tendon;
- Localized fluid collections or hematoma in cases of suspected PM or gastrocnemius rupture (tennis leg);
- Accessory or duplicated tendons, which may be misinterpreted as scar tissue, fibrotic bands, or partial tendon tears;
- The interface between PT and Achilles tendon in patients with medial-sided Achilles pain, which may represent intertendinous friction syndrome.

However, the PT, due to its slender caliber and variability in course, may be misidentified as a pathological structure such as an adherent fibrotic band, degenerative focus, or partial Achilles tendon tear, especially in less experienced hands [17,34,57].

#### 6.3.2. Magnetic Resonance Imaging (MRI)

MRI remains the gold standard for evaluating the PT due to its excellent soft-tissue contrast and multiplanar capability. It allows for:

- Differentiation between PM, the Achilles tendon, and adjacent fascia or neurovascular structures;
- Identification of subtle fluid tracking, suggesting partial rupture, tendinopathy, or peritendinous bursitis;
- Visualization of variant insertions, such as fusion with the Achilles tendon, insertion into the crural fascia, or accessory muscle slips;
- Preoperative mapping before tendon harvesting, especially in cases involving Achilles reconstruction or autograft planning.

MRI is especially valuable in chronic midportion Achilles tendinopathy, where the PT has been implicated as a pain generator, often due to mechanical interference or intertendinous compression [51,52].

#### 6.3.3. Clinical Relevance

Improved imaging-based recognition of the PT and its variants can:

- Prevent misdiagnosis (e.g., mistaking a PT rupture for deep vein thrombosis, Baker’s cyst, or partial Achilles tendon tear);
- Guide treatment decisions, including targeted physical therapy or surgical release of the PT in refractory Achilles pain [65];
- Optimize preoperative planning by ensuring the availability and course of the PT before harvesting;
- Avoid unnecessary interventions or alarm, when the visualized structure is in fact a normal variant [6].

Given the high morphological variability of the PT, such as absence, duplication, or fusion with the Achilles tendon, radiologists, orthopedic surgeons, and sports medicine clinicians must remain vigilant and consistently include the PT in the differential diagnosis of posterior leg pain and Achilles pathology.

## 7. Discussion

The PM has long occupied a controversial position in human anatomy, frequently labeled as vestigial, yet repeatedly recontextualized in surgical, biomechanical, and evolutionary studies. This review consolidates emerging evidence across multiple domains, suggesting that the PM is a morphologically diverse, yet functionally significant, structure.

In the following sections, we synthesize the central themes derived from current anatomical and clinical literature, including the evolutionary ambiguity, architectural variability, and clinical reappraisal of the plantaris. In doing so, we revisit the long-standing question: is the PM a fading evolutionary remnant, or a muscle in transition adapted for new roles?

### 7.1. The Plantaris as a Morphologically Variable but Functionally Relevant Structure

The PM is one of the most variable muscles in the human body, exhibiting a spectrum of origin and insertion types, different courses of the tendon, and even being variably present or absent across populations [5,6]. These variations are often perceived as a sign of redundancy. However, accumulating morphological and histological evidence challenges this notion.

Anatomical studies show that variability in the PM is the rule rather than the exception. Olewnik et al. [5,6,7] proposed a classification system for PM origins and insertions, revealing frequent fusions with the Achilles tendon or insertions into the crural fascia, indicating integration rather than isolation. Case reports of three- and four-headed variants [12,25,26,27] emphasize its architectural plasticity and adaptability.

Histological studies have demonstrated that the PM contains a high density of muscle spindles relative to its size, more than many larger muscles of the posterior leg [48]. This suggests a specialized proprioceptive function, potentially contributing to joint angle monitoring, subtle postural adjustments, or the modulation of tendon tension. The tendon’s long and elastic structure further supports the hypothesis that it may assist in tension distribution during eccentric loading, particularly in the context of the Achilles complex [52].

From an evolutionary standpoint, the PM exhibits significant variation among mammals. In some species, it is robust and active in grasping or climbing functions, whereas in humans, it is reduced or absent in a substantial proportion of the population. This raises the possibility of functional repurposing, where the muscle is retained for proprioceptive or neuromuscular integration rather than gross motor activity [66,67].

In summary, the current evidence indicates that the PM is not merely a phylogenetic remnant. Instead, it should be viewed as a structurally diverse muscle with latent or evolving functional significance. As anatomical science continues to incorporate sensory, biomechanical, and developmental data, the PM may serve as an exemplary model of how variability does not negate function, but rather reflects physiological versatility and adaptation.

### 7.2. Need for Future Research: Biomechanics, Electromyography, and Neuroanatomy

Although recent studies have reignited interest in the PM, substantial gaps remain in our understanding of its actual function in humans. Most available data come from cadaveric dissection, morphological classifications, or isolated clinical reports. In contrast, functional investigations such as in vivo biomechanics, electromyography (EMG), and neuroanatomical mapping are still rarely performed [5,6,48,52].

To clarify the role of the PM in locomotion, further biomechanical studies are necessary. These studies should assess force transmission and tension modulation within the Achilles–plantaris complex, evaluate muscle involvement during eccentric loading and plyometric movement, and incorporate the PM into muscle synergy models related to gait and posture. Such investigations could reveal whether the PM contributes to joint stability or limb energy efficiency, or whether it is contextually activated, for instance during fatigue compensation or dynamic balance adjustments [65].

Electromyography offers a direct means of evaluating neuromuscular activation during movement. However, EMG data for the PM are nearly absent in the current literature. Future studies should aim to determine whether the PM is electrically active during gait, jumping, or stair descent; identify co-activation patterns with the gastrocnemius, soleus, and tibialis posterior; and compare activity between individuals with and without a PT [52].

Histological research has shown a high density of muscle spindles in the PM, suggesting a proprioceptive role [48]. Nonetheless, little is known about its afferent innervation, central neural projections, and participation in reflex arcs. Preliminary data on intramuscular innervation using modified Sihler’s staining have already revealed a complex branching architecture within the PM, including multiple nerve entry points and dense motor endplate zones, indicating proprioceptive specialization [3]. Future studies using neural tracing, immunohistochemistry, or advanced imaging techniques such as diffusion tensor imaging may help clarify the neural pathways involved. This includes whether PM activity supports reflexive postural adjustments or is compensated for by adjacent muscles in cases of agenesis [12].

To resolve the ongoing debate about the PM’s function, interdisciplinary studies combining biomechanics, electrophysiology, and neuroanatomy are essential. Only through such integration can we determine whether the plantaris is truly vestigial or a specialized, underappreciated element of human motor control.

### 7.3. Integration of Fetal Development, Adult Morphology, and Clinical Relevance

A comprehensive understanding of the PM requires a multistage perspective that integrates fetal development, adult anatomical variation, and clinical manifestations. This approach highlights that the morphological diversity observed in adults is not random, but rooted in early developmental patterns.

Waśniewska-Włodarczyk et al. [8,9] demonstrated that the PM is consistently present during the second and third trimesters of fetal life, with distinct muscle bellies and tendons visible as early as 18 weeks of gestation. Notably, the observed insertional and positional variants closely correspond to those documented in adult populations [5,6]. These findings suggest that anatomical variability does not arise postnatally, but reflects a stable, genetically programmed polymorphism modulated by epigenetic and mechanical influences during later gestation.

Understanding embryogenesis has direct implications for adult anatomy and surgical practice. Variants such as duplicated tendons or non-calcaneal insertions, commonly seen in fetal specimens, match findings in adult surgeries [10,32]. Embryological data can help distinguish true agenesis from hypoplasia or atrophy and inform preoperative planning by anticipating variability in tendon length, course, or insertion site [5].

Developmental insights also enhance clinical interpretation. They may help clinicians anticipate unexpected intraoperative findings, distinguish benign anatomical anomalies from pathological ones on imaging, and better understand pain patterns or dysfunctions. This is particularly relevant to midportion Achilles tendinopathy, where unusual plantaris insertions may lead to increased friction or irritation [52].

The PM illustrates how developmental biology informs adult anatomy and clinical behavior. Rather than representing a degenerate remnant, its diverse morphologies reflect conserved developmental programs. Further research should focus on mapping the development structure function triad with translational applications in surgery, diagnostics, and rehabilitation.

### 7.4. Limitations of the Review

Although this review offers a comprehensive synthesis of the plantaris muscle across multiple domains, several limitations must be acknowledged. First, the lack of functional studies, particularly electromyography (EMG) and in vivo biomechanical analysis, limits the ability to definitively assess the PM’s role in movement and proprioception. Second, comparative anatomical data among mammals often stem from small sample sizes or isolated case studies, reducing generalizability. Third, variations in dissection techniques and classification criteria across studies introduce methodological heterogeneity, which may impact anatomical interpretation. Finally, while histological evidence supports a sensory function, direct neuroanatomical mapping of afferent pathways remains scarce and requires further investigation.

## 8. Conclusions

Collectively, the current evidence challenges the long-held view of the PM as a vestigial structure. Embryological data confirm its consistent development, while adult morphology reveals complex and stable variants, many of which have clinical and biomechanical relevance. Comparative anatomy further supports the idea that the PM is selectively retained or lost across species depending on locomotor needs. In humans, histological and anatomical features suggest a proprioceptive function rather than primary force generation.

Clinically, the PM influences both imaging interpretation and surgical planning, especially in tendon grafting and management of Achilles tendinopathy, highlighting its growing relevance. Moreover, the diversity of its anatomical forms underscores the necessity of tailored diagnostic and therapeutic strategies.

We propose that the PM should not be considered a degenerating remnant, but rather a functionally transforming muscle, potentially evolving toward sensorimotor integration in bipedal locomotion. Future research should focus on functional validation through electromyography, biomechanical analysis, and neuroanatomical tracing to fully elucidate its modern role in human physiology.

## Figures and Tables

**Table 1:** Plantaris muscle proximal attachment types (Olewnik et al. [5]).

Type	Anatomical Description	Clinical Note	Frequency (%)
Ia	Originates in the lateral femoral condyle, knee joint capsule, and lateral head of the gastrocnemius muscle.	Most common configuration; proximity to posterolateral joint capsule and neurovascular structures may increase surgical relevance.	39.8
Ib	As in Ia, with additional attachment to the popliteal surface of the femur.	May increase mechanical coupling with posterior femoral structures; relevant in extended posterior approaches to the knee.	8.6
II	Originates in the knee joint capsule and lateral gastrocnemius head (indirectly connected to the lateral femoral condyle).	Lacks direct bony attachment; may reflect embryological linkage with gastrocnemius and contribute to complex soft-tissue entwinement.	25.0
III	Originates in both the lateral femoral condyle and the knee joint capsule.	Classical posterolateral pattern; relevant in cases of capsular or bony entrapment syndromes.	10.15
IV	Originates in the lateral femoral condyle, knee joint capsule, and iliotibial band.	Potentially associated with iliotibial band syndrome (ITBS); structural traction may alter lateral knee mechanics.	6.25
V	Originates solely in the lateral femoral condyle.	Simple and compact; may be misidentified during arthroscopic procedures due to lack of fascial or muscular overlap.	8.6
VI	Rare and unclassified cases. Includes: Double plantaris—main PM from lateral femoral condyle, knee capsule, iliotibial band; accessory from iliotibial band, distal fusion with semimembranosus tendon and oblique popliteal ligament. Bifurcated plantaris—lateral head from lateral GM; medial head from knee capsule beneath GM; both converge into one tendon.	Variants may mimic soft-tissue tumors or compress neurovascular structures. Recognition is essential during surgery or imaging.	1.6

**Table 2:** Comparative overview of plantaris muscle origin classifications.

Author	Sample Size	Described Origins	Formal Classification	Clinical Relevance
Cummins & Anson [19]	200 limbs	Lateral supracondylar ridge; fibrous extensions to capsule and gastrocnemius	No	Foundational anatomical observation
Nayak et al. [18]	52 lower limbs	Lateral supracondylar ridge, lateral condyle, joint capsule	Yes (3 types)	Descriptive and population-specific data
Freeman et al. [14]	46 limbs	Oblique popliteal ligament; shared fibers with gastrocnemius	No	Suggests accessory and blended origins
Olewnik et al. [5]	142 lower limbs	Six distinct types (Type I–VI) with subtypes for Type I	Yes (detailed classification)	Clinically applicable; guides imaging and surgical procedures

**Table 3:** Plantaris tendon insertion types and clinical implications.

Classification/Author	Anatomical Characteristics	Clinical Implications
Olewnik et al.—Type I	Fan-shaped insertion on the calcaneal tuberosity, medial to Achilles tendon	Risk of irritation due to close proximity to Achilles tendon
Olewnik et al.—Type II	Band-shaped insertion directly into the medial Achilles tendon, common paratenon	High potential for mechanical irritation; clinically significant
Olewnik et al.—Type III	Insertion anterior to the Achilles tendon on the calcaneus	May contribute to compression or friction syndromes
Olewnik et al.—Type IV	Insertion into deep crural fascia, no bony attachment	Generally asymptomatic; important for graft harvesting
Olewnik et al.—Type V	Broad insertion wrapping posterior and medial Achilles tendon	Increased contact with Achilles; risk in repetitive stress
Olewnik et al.—Type VI	Insertion near the flexor retinaculum of the tarsal canal	Relevant for differential diagnosis; rarely symptomatic
Olewnik et al.—Type VII	Bifid tendon with multiple distal insertions	May mimic multiple insertion syndromes; surgical relevance
Olewnik et al.—Type VIII	The PT ends within the soleus muscle	Usually asymptomatic; important for harvesting procedures
van Sterkenburg—Types 1–3	Various medial and fan-shaped insertions on calcaneus and Achilles	Associated with midportion Achilles tendinopathy
van Sterkenburg—Type 9	Insertion into the crural fascia	Typically asymptomatic; anatomically relevant for diagnosis
dos Santos—Types I–III	Medial and anteromedial insertion or merging with Achilles	May cause confusion on imaging; associated with pain
Cummins & Anson—medial/fused	Medial insertion onto calcaneus or blending with Achilles	Described early risk variants; limited detail on morphology

**Table 4:** Presence of plantaris muscle in selected mammals.

Order	Species (Example)	Scientific Name	Presence of PM	Functional Notes
Primates	Human	Homo sapiens	Variable (7–20% agenesis)	Possibly proprioceptive; frequently absent or fused (Daseler & Anson, 1943 [1]; Olewnik et al., 2018 [6])
Primates	Chimpanzee	Pan troglodytes	Present	Well-developed; involved in climbing and ankle control (Diogo & Wood, 2011 [39])
Carnivora	Dog	Canis familiaris	Present	Contributes to propulsion and extension of the tarsal joint (Evans & de Lahunta, 2012 [41])
Carnivora	Cat	Felis catus	Present	Functional; synergistic with gastrocnemius and superficial digital flexor (Evans & de Lahunta, 2012 [41])
Perissodactyla	Horse	Equus caballus	Absent	Functionally replaced by SDFT and gastrocnemius; no independent PM (Getty, 1975 [43])
Artiodactyla	Cow	Bos taurus	Absent	Absent; locomotion driven by tendons and ligaments with minimal musculature (Dyce et al., 2010 [42])

## Data Availability

All data supporting the findings of this study are available from the corresponding author upon reasonable request.
